# Band Structure of Topological Insulator BiSbTe_1.25_Se_1.75_

**DOI:** 10.1038/s41598-017-04985-y

**Published:** 2017-07-04

**Authors:** H. Lohani, P. Mishra, A. Banerjee, K. Majhi, R. Ganesan, U. Manju, D. Topwal, P. S. Anil Kumar, B. R. Sekhar

**Affiliations:** 1 Institute of Physics, Sachivalaya Marg, Bhubaneswar, 751005 India; 2 Homi Bhabha National Institute, Training School Complex, Anushakti Nagar, Mumbai, 400085 India; 3 Indian Institute of Science, Bangalore, 560012 India; 4 CSIR-Institute of Minerals and Materials Technology, Bhubaneswar, 751005 India

## Abstract

We present our angle resolved photoelectron spectroscopy (ARPES) and density functional theory results on quaternary topological insulator (TI) BiSbTe_1.25_Se_1.75_ (BSTS) confirming the non-trivial topology of the surface state bands (SSBs) in this compound. We find that the SSBs, which are are sensitive to the atomic composition of the terminating surface have a partial 3D character. Our detailed study of the band bending (BB) effects shows that in BSTS the Dirac point (DP) shifts by more than two times compared to that in Bi_2_Se_3_ to reach the saturation. The stronger BB in BSTS could be due to the difference in screening of the surface charges. From momentum density curves (MDCs) of the ARPES data we obtained an energy dispersion relation showing the warping strength of the Fermi surface in BSTS to be intermediate between those found in Bi_2_Se_3_ and Bi_2_Te_3_ and also to be tunable by controlling the ratio of chalcogen/pnictogen atoms. Our experiments also reveal that the nature of the BB effects are highly sensitive to the exposure of the fresh surface to various gas species. These findings have important implications in the tuning of DP in TIs for technological applications.

## Introduction

Discovery of the new quantum state of matter called topological insulators (TI) has attracted world wide interest due to their exotic properties which are manifestations of a non-trivial band topology^1, 2^. TIs have insulating bulk and conducting edges due to the presence of some peculiar surface states (SSs). These SSs are spin non-degenerate with a unique property of spin momentum locking which results from the strong spin-orbit coupling (SOC) effects in combination with time reversal symmetry. It has been theoretically predicted that these SSs host many interesting properties like, Dirac fermion^1, 3^, magnetic monopole^4^ and Majorana bound state at the vortex in superconducting regime^5, 6^. Strong immunity of these SSs to Anderson localization and backscattering in presence of non-magnetic impurities have tremendous technical advantages, especially for functional applications like spintronic devices and quantum computers^7, 8^. Furthermore, tunability of the crossing point of the topological SSs, called the Dirac point (DP) by chemical doping, is another aspect important from such technological point of view^9–12^. In the known Bi and Sb based binary TIs the DP and the SSs are often obscured by contributions from bulk states. Tetradymite Bi_2_Te_3_ Se which is isostructural to the prototypical TIs Bi_2_Se_3_ and Bi_2_Te_3_ has been found to be suitable for such tuning of the DP within the bulk band gap owing to its relatively large bulk resistivity^13^. The resistivity can be optimized in the Sb doped quaternary alloy Bi_2−*x*_Sb_*x*_Te_3−*y*_Se_*y*_ by changing the ratio of the pnictogen (Bi and Sb) and chalcogen (Se and Te) atoms without disturbing its crystallinity. In this compound, topological nature with different bulk resistivity has been experimentally observed in a wide range of x and y combinations^14^. Thus, Bi_2−*x*_Sb_*x*_Te_3−*y*_Se_*y*_ provides an ideal platform to study the nature of topological surface states by tuning the Dirac node through controlling the proportion of chalcogen/pnictogen atoms.

Recently, quantum hall effect (QHE)^15^ and scanning tunnelling spectroscopy (STS)^16^ studies have been used to confirm the topological characters of BiSbTeSe_2_ and Bi_1.5_Sb_0.5_Te_1.7_Se_1.3_. Tunability of the Dirac cone also has been observed in some of the compositions of Bi_2−*x*_Sb_*x*_Te_3−*y*_Se_*y*_ by using angle resolved photoelectron spectroscopy (ARPES) measurements^17, 18^. Furthermore, the low bulk carrier density in these materials allows electrostatic gating of the chemical potential letting a strong control over electrical transport properties which is vital for applications^15, 19, 20^. While most of the reported studies were focussed on the tunability by chemical doping or adding layers of other elements on the surface of TIs, the drifting of the topological surface state bands (SSBs) and DP with aging of the surface which are also important for device applications^21^, has not been addressed adequately in this family of TIs^9, 10^. In this paper, we present a detailed study of the electronic structure and aging effects of BiSbTe_1.25_Se_1.75_(BSTS) using ARPES in conjunction with density functional theory (DFT) based calculations. In the ARPES data, we observe topological character of the SSBs and a warping of the Fermi surface (FS). These results are consistent with our calculated SSBs which fall within the region of bulk band gap of BSTS. In addition, we find pronounced effects of aging due to band bending (BB) and which are relatively stronger in this compound in comparison to Bi_2_Se_3_. Effects of the BB are enhanced due to the high adsorption of residual gases at low temperatures. Furthermore, experiments performed with constant dosing of different gases show that the BB effects are highly sensitive to the gas species.

## Results and Discussion

**Figure 1:**
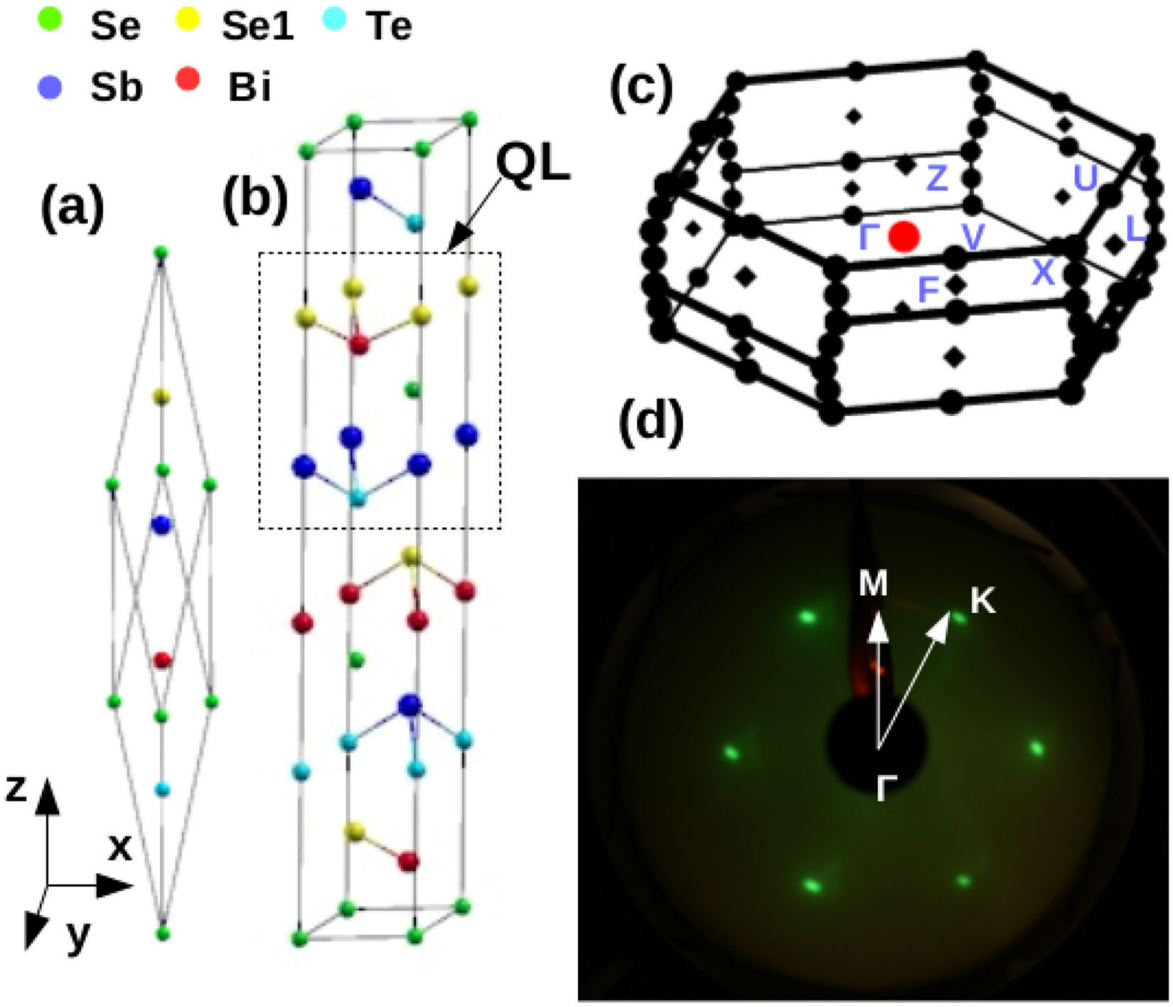
(**a** and **b**) show the primitive and hexagonal unit cells of BiSbTeSe_2_ respectively. The dotted box encloses the structure of five atomic layers which is called quintuple layer (QL). (**c**) Brillouin zone of the primitive unit cell where high symmetry k-points are marked. (**e**) LEED spots depict the hexagonal symmetry of the surface BZ of BSTS where the high symmetry k-directions Γ-K and Γ-M are marked.

Figure 1(a) shows the primitive unit cell of BiSbTeSe_2_ which has a rhombhohdral symmetry. This structure can also be depicted as a hexagonal unit cell as shown in Fig. 1(b). Basic building block of this structure is the so called quintuple layer (QL) consisting of five atoms arranged in an order Se1-Bi-Se-Sb-Te. The Bi(Se1) atoms are connected to the Sb(Te) atoms with the center of inversion symmetry at the Se atom site. The structure of BiSbTeSe_2_ is similar to that of Bi_2_Se_3_. Substitution sites of Sb and Te in Bi_2_Se_3_ to build the BiSbTeSe_2_ structure for our calculations were chosen by considering the total energy minimization among various possible structures. Figure 1(c) shows the Brillouin zone (BZ) of the primitive unit cell where the high symmetry k-points are marked. In Fig. 1(d) we have shown a low energy electron diffraction (LEED) pattern from our BSTS crystal depicting the hexagonal symmetry of the surface BZ. The BSTS crystal cleaves along the (111) crystal plane and scanning tunnelling microscopy experiments have shown that the terminated plane has Te/Se atoms on it ref. 16.

**Figure 2:**
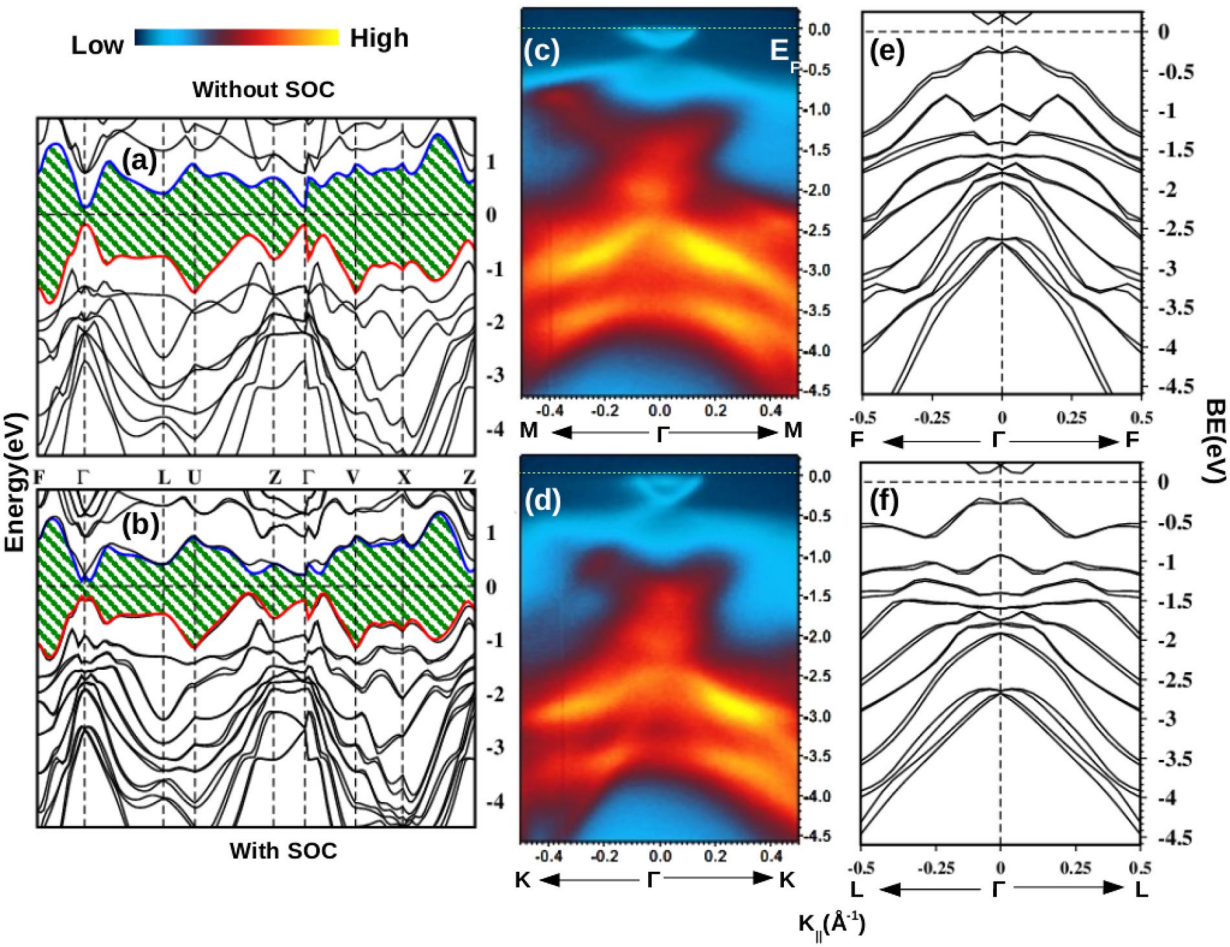
(**a** and **b**) show the bulk band structure plots of BiSbTeSe_2_ without and with inclusion of SOC effects respectively. (**c** and **d**) Show the ARPES images of BSTS along the Γ-M and Γ-K directions of the surface BZ respectively. (**e** and **f**) Show the bulk bands of (**b**) along the Γ-F and Γ-L directions respectively.

Figure 2(a and b) show the bulk band structure of nominal BiSbTeSe_2_ composition without and with inclusion of SOC effects respectively. In both the cases, valence band(VB) and conduction band(CB) states are well separated in the energy scale. However, the SOC effects induce a small splitting in the bands along various k-directions. The band gap is ~0.45 eV at the Γ point in SOC included case (Fig. 2(b)) which is higher than the value 0.3 eV found in Bi_2_Se_3_^22^. Structure of the top most VB(red) and the lowest CB(blue) along the F-Γ-L direction indicate a band inversion at the Γ point after incorporating the SOC effects. This results in a non-trivial value of the Z_2_ invariant in this system^23^. In Fig. 2(c and d) the ARPES intensity plots of BSTS, which has a slightly different stoichiometry (BiSbTe_1.25_Se_1.75_) from the nominal composition (BiSbTeSe_2_), are presented. These plots are taken along the Γ-M and Γ-K directions of the surface BZ by using 31 eV photon energy respectively. Among the various bands seen in the VB region, the deeper lying bands with binding energy (BE) in the range E_*b*_ = −0.5 to −4.5 eV show a highly dispersive nature. A cone shaped distribution of low intensity is clearly visible in the vicinity of the E_*f*_ around the Γ point which is absent in the calculated band structure. The cone is formed by the topological SSBs in the bulk band gap region of the material. Although, inconsequential to the results of this study, a closer look at the data in the Fig. 2(c) will reveal the existence a small energy gap near the tip of the cone which could be due to some possible misalignment of the angular position of the sample to the perfect Γ-M direction during the data collection. Nevertheless, in order to have a better comparison, raw data of the bulk bands along the Γ-F and Γ-L directions (Fig. 2(b)) which correspond to the Γ-M and Γ-K directions of the surface BZ, are plotted adjacent to the ARPES images in Fig. 2(e and f) respectively. Along both the directions, calculated bands placed at higher BE show a fair resemblance to the corresponding intensity pattern observed in the ARPES images.

**Figure 3:**
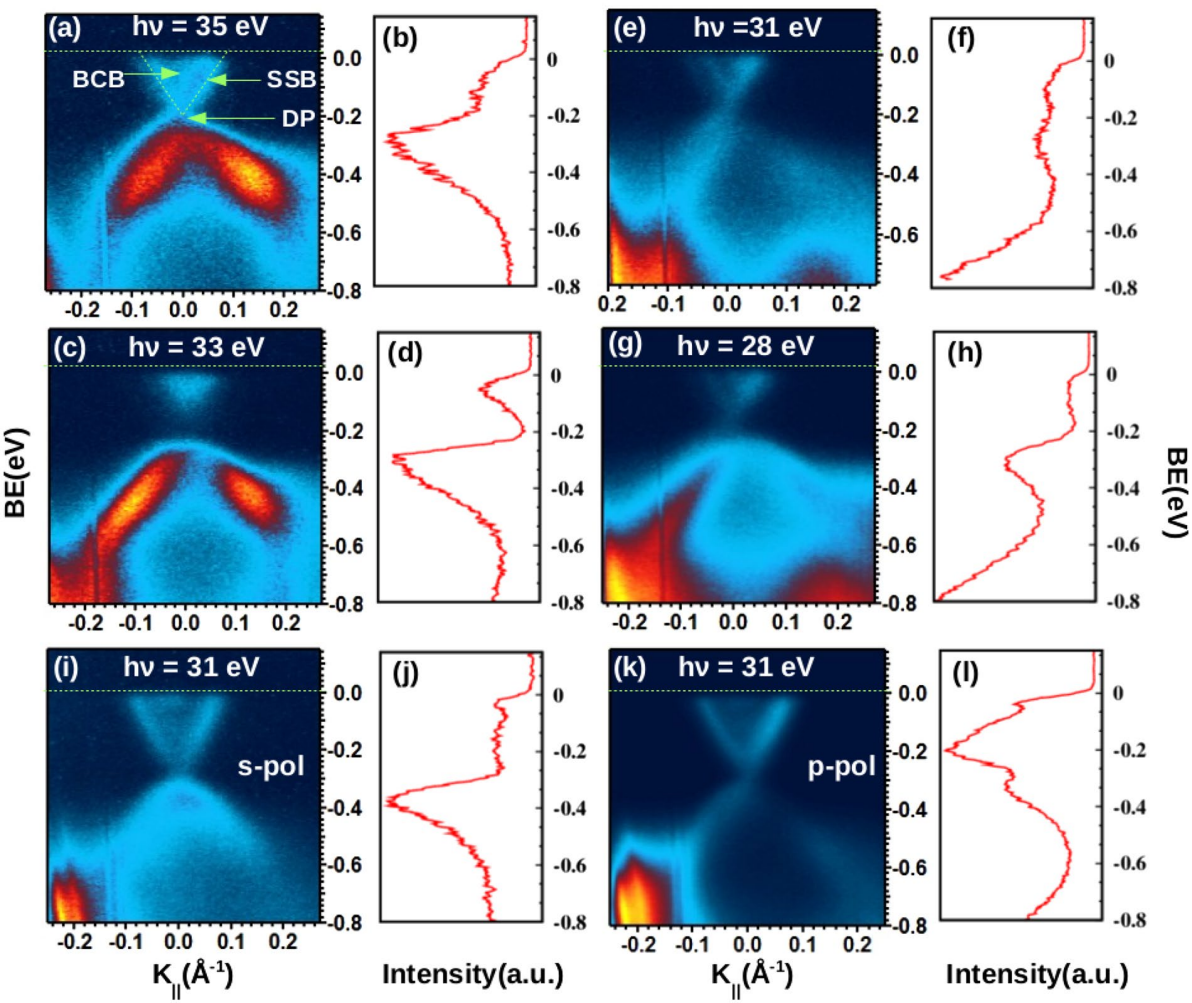
(**a**,**c**,**e** and **g**) correspond to the near E_*f*_ ARPES images taken at photon energy 35, 33, 31 and 28 eV respectively. (**b**,**d**,**f** and **h**) Show EDC spectra corresponding to the images in (**a**,**c**,**e** and **g**) respectively. Intensity map using s and p-polarized photon energy of 31 eV are shown in (**i** and **k**) respectively. EDC spectra of these images are shown in (**j** and **l**) respectively.

Figure 3(a) shows the near E_*f*_ region of ARPES plot taken by using 35 eV photon energy. The two SSBs are clearly visible exhibiting almost a linear dispersion. Intensity between these two SSBs indicates the presence of the bulk conduction band (BCB) states occupied due to n-type intrinsic impurities and defects. On the other hand, lower part of the Dirac cone strongly overlaps with the bulk valence band (BVB) states which form a high intensity region at ~E_*b*_ = −0.4 eV. In order to identify the position of the DP, the corresponding energy density curve (EDC) of the image shown in Fig. 3(a) is plotted in Fig. 3(b). This EDC is taken at the Γ point with k width of ±0.02 Å^−1^. As is clear from this spectra the DP appearing at E_*d*_ ~ −0.2 eV is obscured by the emission from the BVB states. In order to confirm the surface nature of the SSBs, ARPES images were collected at different photon energies as shown in Fig. 3(a,c,e and g) which correspond to images taken at photon energy 35, 33, 31 and 28 eV respectively. EDCs(at the Γ point with k width of ±0.02 Å^−1^) corresponding to these intensity plots are presented in Fig. 3(b,d,f and h) respectively. The BVB gets modified sharply with the variation in photon energy indicating the bulk nature of these higher BE bands while the shape of near E_*f*_ SSBs (upper part of Dirac cone) remains unaffected confirming the surface state character. The slight variation in the intensity of these SSBs is due to the difference in the matrix elements involved in the photoemission process^3^. It should be noted that the SSBs show some significant changes close to the DP. The EDC spectra of 33 eV photon energy (Fig. 3(d)) shows an apparent opening of a gap in the SSBs in the vicinity of the DP (E_*d*_ = −0.2 eV), unlike the case of 35 eV photon energy (Fig. 3(b)). Similarly, the spectral weight near ~−0.2 eV BE in the EDC of 31 eV (Fig. 3(f)) also shows differences compared to that taken with 28 eV (Fig. 3(h)) photon energy. These results show that the SSBs are not of pure 2D character in this compound. The SSBs which mainly form the lower part of the Dirac cone hybridize with the BVB states and therefore acquire a partial 3D character. Origin of these hybridized states could be the impurities or defects in the system as suggested in a theoretical model for finite bulk band gaps by Black-Schaffer *et al*.^24^. Experimental realization of such impurity induced gap opening in the SSBs at the DP has been recently reported by Sanchez-Barriga *et al*. in their detailed ARPES study of (Bi_1−*x*_Mn_*x*_)_2_ Se_3_ system^25^. Figure 3(i and k) show the ARPES plot taken with s and p-polarized light of photon energy 31 eV respectively and adjacent Fig. 3(j and l) display the EDC(at the Γ point with k width of ±0.02 Å^−1^) corresponding to them. In both the cases the linearly dispersive SSBs are clearly seen. However, the intensity of the BVBs at ~E_*b*_ = −0.5 eV got drastically reduced in the p-polarized case compared to the s-polarized. These changes are also visible in the spectral features of their EDCs (Fig. 3(j and l)) showing the different orbital characters of these bands.

**Figure 4:**
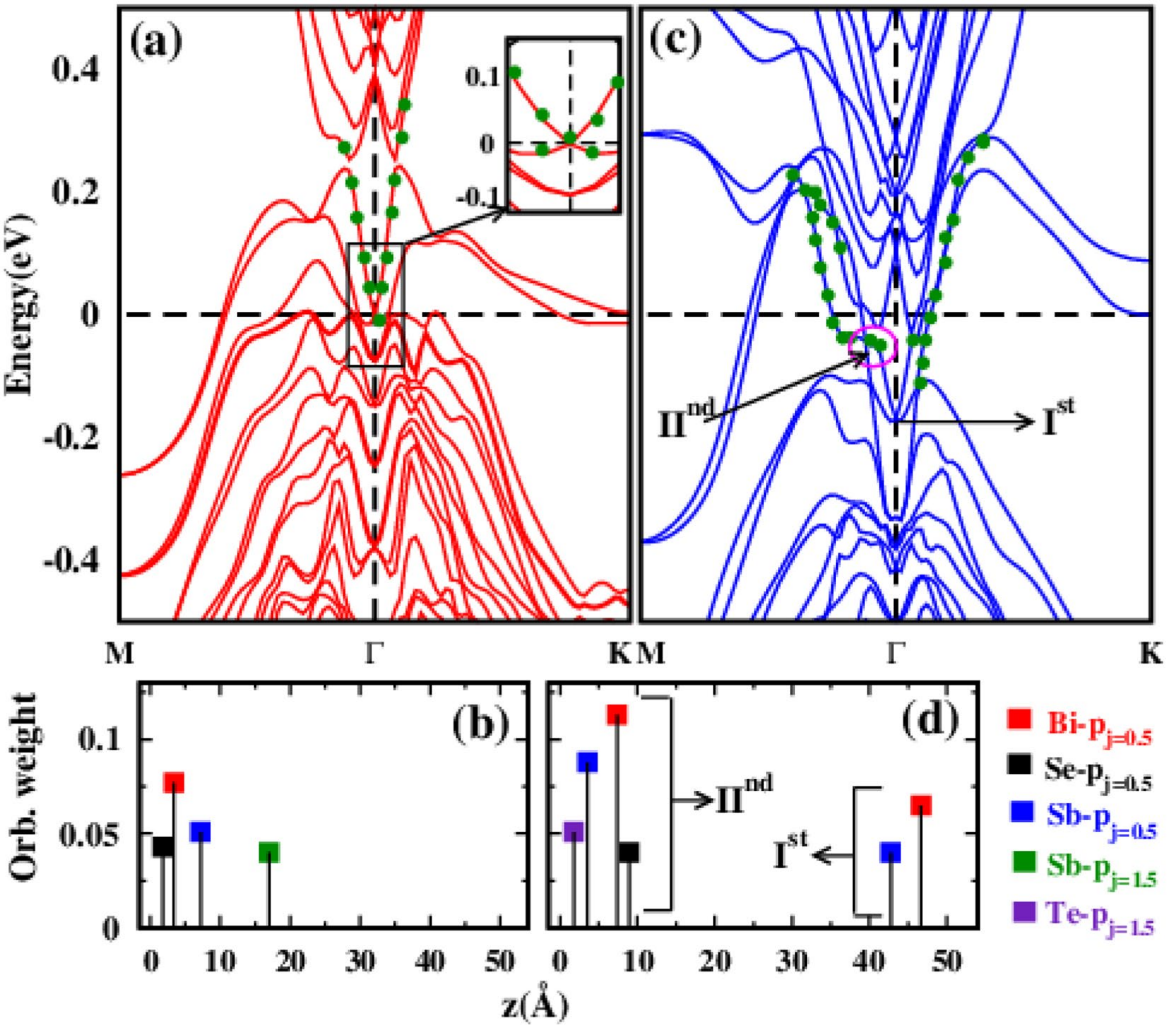
(**a** and **c**) show calculated bands of Se1 and Te terminated face of 6QL slab geometry of BiSbTeSe_2_ respectively. (**b**) Weight of atomic orbitals mainly contributing to the ‘V’ shaped band (enclosed in the black box) at the Γ point in Se terminated plain with respect to their distances from the surface. (**d**) Similar contribution to V shaped band at the Γ point (I^*st*^) and slightly away from the Γ point (II^*nd*^) in Te terminated face.

Characteristics of the SSBs have been investigated by performing surface state calculations on the (111) crystal plane of BiSbTeSe_2_. In Fig. 4(a) bands (red) of 6QL slab structure with Se1 terminated face are plotted along the M-Γ-K direction of the surface BZ. It can be seen that two bands falling into the shape of ‘V’ are observed just above the E_*f*_ around the Γ point. Here, green dots represent the orbital contribution coming from the atoms present in the topmost QL. Crossing of these Dirac like SSBs occurring at E_*f*_ is more clearly visible in the inset. In order to figure out the origin of these ‘V’ shaped bands, orbital projection weight of majorly contributing atoms to these bands at the Γ point with respect to their distances from the surface (z) are plotted in Fig. 4(b). Figure 4(b) shows clearly that the orbital character originates primarily from the atoms close to the surface rather than the bulk region and the same character also persists in the nearby k-points of the Γ point. This further confirms the surface state nature of these bands. These results show a qualitative similarity to the previously reported SSBs of BiSeTe_2_^23^. The ‘V’ shaped bands show a high resemblance to the intensity pattern of SSBs observed in the ARPES data (Fig. 3(a)), though there is slight mismatch in the Fermi position. The mismatch is possibly due to the intrinsic n-doping in the sample which raises the E_*f*_ level in the experimental data. The other possibility of Te terminated surface has also been examined and bands (blue) of 6QL slab of this geometry are shown in Fig. 4(c). In this case, the ‘V’ shaped band around the Γ point (I^*st*^ region) deviates from linearity as it moves away from the Γ point (II^*nd*^ region). Position of the tip of this V shaped band (180 meV) is quite below the E_*f*_, unlike the case of Se terminated face (Fig. 4(a)). This energy position differs from the experimentally observed BVB (80 meV) of freshly cleaved BSTS (see Fig. 1(e) of Supplementary note). In addition, the orbital weight of these bands at region I^*st*^ and II^*nd*^ are dominated by atomic orbitals placed in the bulk and surface sites respectively as is clear from the Fig. 4(d). Probably, this large mixing of bulk and surface characters leads to the deviation in the dispersion of this band. This different origin of the ‘V’ shaped band around the Γ point from bulk and surface in the Te and Se termination cases show that the SSBs are sensitive to the atomic composition of the surface.

**Figure 5:**
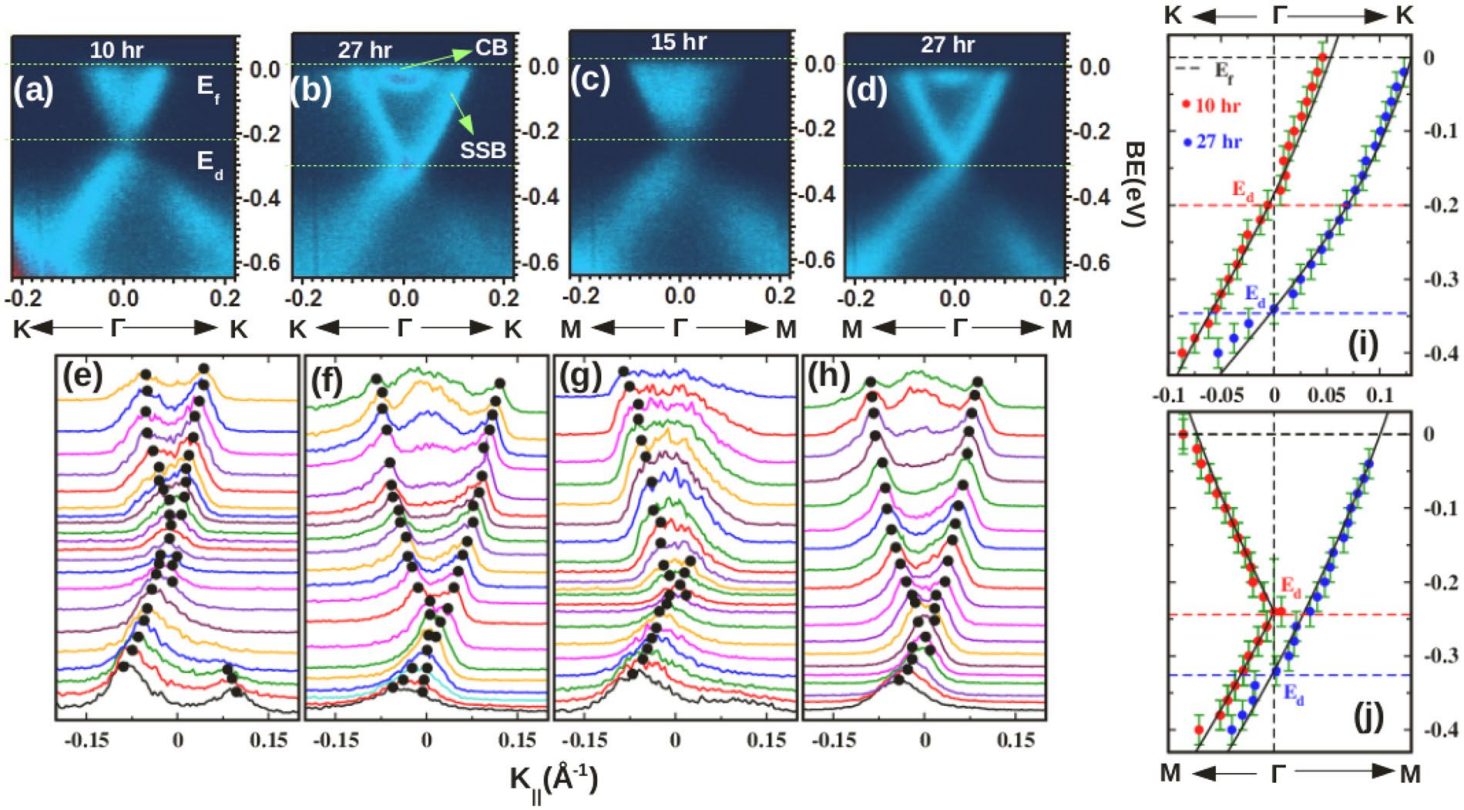
(**a** and **b**) depict the ARPES intensity plots taken at 31 eV photon energy along the Γ-K direction taken ~10 and 27 hrs. after the sample cleaving. (**c** and **d**) Show the same images along Γ-M direction collected at different time intervals from the cleaving. (**e**–**h**) Show MDC spectra extracted from the images (**a**–**d**). The dispersion relation between E and k estimated from the MDC plots are fitted to the calculated values obtained from the model Hamiltonian^28^ along the Γ-K (**i**) and the Γ-M (**j**) directions.

As mentioned before, the tunability of the DP within the bulk band gap of the system is an advantage of BSTS important from the technological point of view which could be achieved by chemical doping. Intimately related to this is the observed gradual shifting of the DP with adsorption of gases or even the elapse of time in ultra high vacuum after the crystal cleaving. This shifting of DP is caused by the band bending which has been observed previously in various TIs, like Bi_2_Se_3_, Bi_2_Te_3_^26, 27^. We present our observations of BB effects on the SSBs in Fig. 5, where 5(a) and (b) show ARPES images along the Γ-K direction collected ~10 and 27 hours after the cleaving. As can be seen from Fig. 5(b), a significant shift of ~0.14 eV is observed in the position of E_*d*_ in comparison to that in Fig. 5(a). Further, the filled CB states in the nearby E_*f*_ region around the Γ point are clearly demarcated from the SSBs and an arc shaped structure is seen in these CB states which could be a signature of two dimensional electron gas (2DEG) character arising due to the strong BB, like in Bi_2_Se_3_^26^. Similarly, shift of E_*d*_ and appearance of distinct SSBs and CB states can also be found in the ARPES images Fig. 5(c) and (d) which were collected along the Γ-M direction at different time intervals after the cleaving. Figure 5(e–h) show plots of momentum density curves (MDC) extracted from the ARPES images of Fig. 5(a–d) respectively, where the linear dispersion of the MDC peaks is clearly seen. An energy dispersion relation of the SSBs can also be obtained from the model Hamiltonian approach proposed by Fu^28^ by the following relation.

1 $$\begin{array}{c}{E}_{\pm }(\vec{k})={E}_{0}(k)\pm \sqrt{{\nu }_{k}^{2}{k}^{2}+{\lambda }^{2}{k}^{6}{\cos }^{2}\mathrm{(3}\theta )}\\ {\rm{here}},\,{E}_{0}={k}^{2}/\mathrm{(2}{m}^{\ast });\quad {\nu }_{k}={\nu }_{0}\mathrm{(1}+\alpha {k}^{2})\end{array}$$

where E_±_ corresponds to the energy of the upper and lower band, E_0_(k) generates particle-hole asymmetry, m* denotes effective mass, and *θ* indicates the azimuthal angle of momentum $\vec{k}$ with respect to the x-axis (Γ-M direction). *λ* is a parameter for the hexagonal warping. *ν*_0_ is the Dirac velocity which is modified to *ν*_*k*_ after including a second order correction parameter (*α*) to the Dirac velocity in the k.p Hamiltonian. The peak positions measured from the MDC plots along the Γ-K and Γ-M directions are fitted to the E-k dispersion relation of the SSBs obtained from the Eq. (1) in Fig. 5(i and j) respectively. The calculated bands nicely fit near the DP while a slight deviation can be seen in the regions away from the DP where the states of BVB and BCB are predominant. Parameters used for the fitting are tabulated in Table 1 which shows that *ν*_0_ reduces significantly along the Γ-K direction after 27 hrs. from the cleaving in comparison to the 10 hrs. cleaving case. On the other hand, warping strength, defined as $\sqrt{\lambda /{\nu }_{0}}$ remains almost constant under the influence of BB. The estimated value of warping strength ($\sqrt{\lambda /{\nu }_{0}}$ = 6.8) is intermediate between the value found in Bi_2_Se_3_ and Bi_2_Te_3_^29^. This result clearly establishes that FS warping and associated out of plane spin polarization can be controlled by the ratio of chalcogen/pnictogen atoms in Bi/Sb based TIs.

**Table 1:** Parameters of calculated SSBs obtained after fitting with the E-k dispersion relation Eq. (1) estimated from the ARPES data along the Γ-K and the Γ-M directions.

Time(hr.)	1/2 m* (eV.Å^3^)	*ν*_0_(eV.Å)	*α*(eV.Å^3^)	*λ*(eV.Å^3^)
**Γ-K**				
10	7	3.0	5	130
27	1.8	1.8	5	80
**Γ-M**				
15	5	2.85	5	—
27	4	2.65	5	—

**Figure 6:**
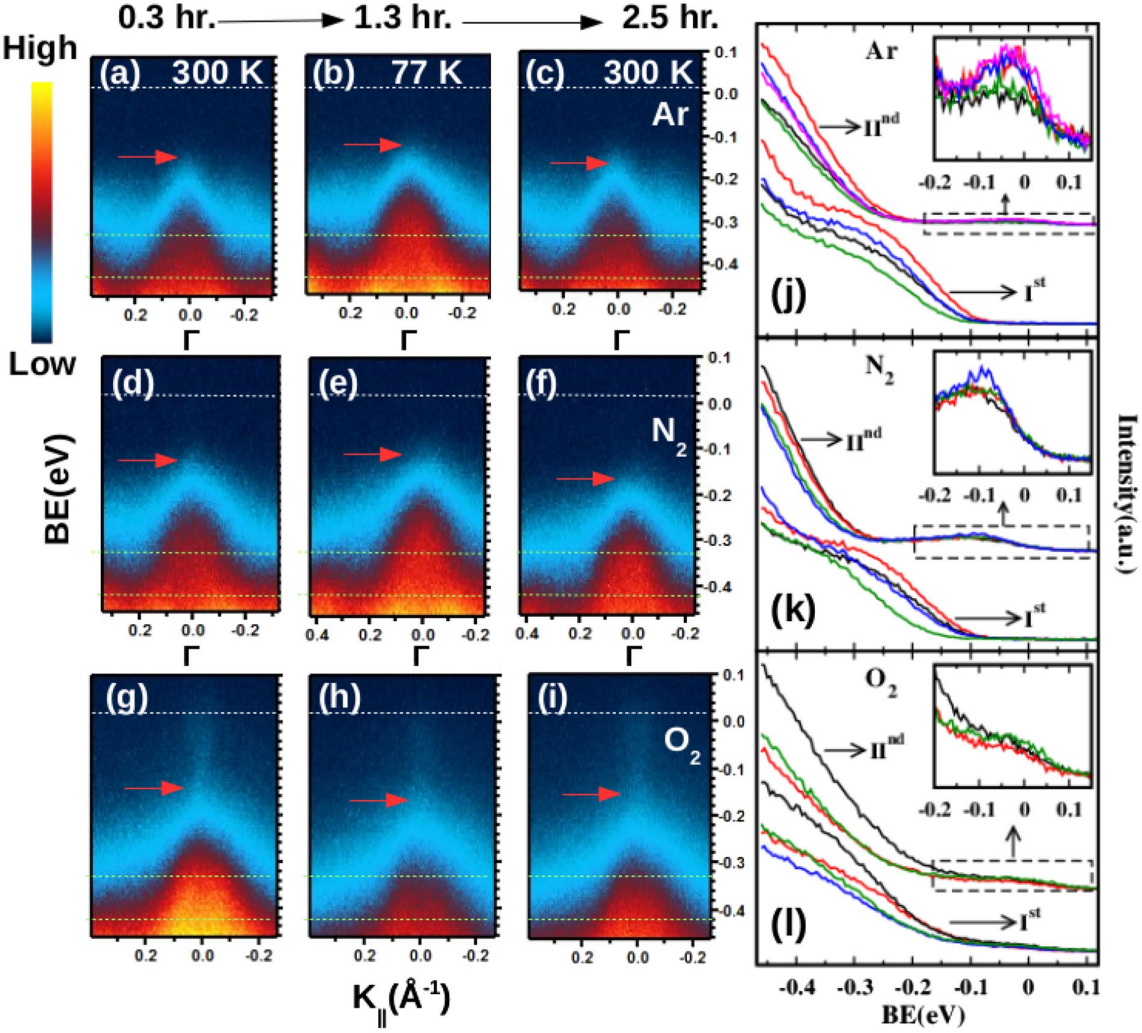
(**a,b** and **c**) show ARPES images taken at 300 K-77 K-300 K respectively just after the cleaving (I^*st*^ thermal cycle) under constant Ar exposure. (**d**–**f** and **g**–**i**) display the ARPES images of the I^*st*^ thermal cycle performed under constant dosing of N_2_ and O_2_ gases respectively. (**j**,**k** and **l**) correspond to the EDC (taken around the Γ point) plots of thermal cycling under the exposure of Ar, N_2_ and O_2_ gases respectively, where the inset of each plot shows the enlarged view of the near E_*f*_ region. Different colours (black → red → green → blue → magenta) of the EDC spectra represent various stages (300 K → 77 K → 300 K → 77 K → 300 K) of thermal cycling respectively.

Further experiments were performed to understand the BB effect by using a laboratory HeI (21.2 eV) photon source in combination with a Scienta R3000 electron energy analyzer. It has earlier been proposed that the BB in TIs originate from accumulation of additional charges at the surface and these extra charges arise due to Se vacancies present in the bulk as well as those created at the surface in the process of surface cleaving^30, 31^. Moreover, adsorption of residual gases further changes the charge distribution at the surface^10–12^. In order to understand the role of adatoms in BB, we undertook the ARPES measurements over a cycle of temperatures, 300 K-77 K-300 K. Since, the adsorption of residual gases is faster at low temperatures the effect of BB is expected to enhance at low temperatures. This view is fairly supported by our thermal cycling ARPES data taken at different time intervals under constant exposure of Ar, N_2_ and O_2_ gases. In first panel, Fig. 6(a,b and c) show the ARPES images taken at 300 K-77 K-300 K respectively just after the cleaving (I^*st*^ thermal cycle) in the Ar environment. Similarly, second (Fig. 6(d–f)) and third (Fig. 6(g–i)) panels display the ARPES images of the I^*st*^ thermal cycle performed under constant dosing of N_2_ and O_2_ gases respectively. In Fig. 6(b), a marginal shifting of the BVB maximum (marked with red arrow) is observed towards the E_*f*_, though it was recorded at a later time in compared to Fig. 6(a). This result of BB is contrary to the behavior observed under the ultra high vacuum conditions. It indicates that the Ar adatoms act like an electron acceptor compensating the Se vacancy induced downward BB and thereby lead to a small upward shifting of the BVB. This inference is supported by the relatively large downward shift of the BVB due to the gas desorption in the next 300 K annealed data (Fig. 6(c)). These changes are more clearly visible in the higher BE region (between the two red dotted lines). Similar characteristics of hole doping are also seen under the exposure of N_2_ gas (Fig. 6(d–f)). However, the dosing of O_2_ gas gives rise to an opposite BB effect *i. e*. features of n-doping as clear from Fig. 6(g–i). Further in this case, a faint feature of the SSBs appears quite early at the top of the BVB, unlike the other gas exposure cases after the cleaving. This probably is linked to the higher adsorption of O_2_ gas which accelerates the BB. These changes are also compared in the EDC (taken around the Γ point of k width of ±0.02 Å^−1^) plots Fig. 6(j,k and l) which correspond to the first, second and third panels respectively. Data sets of thermal cycling performed ~0:30 and 24:00 hrs. after the cleaving are marked I^*st*^ and II^*nd*^ respectively. In Fig. 6(j), a sharp reduction is observed in the intensity of initial 300 K spectra (black) in comparison to the 77 K spectra (red) which again raise and fall in the next 77 K (blue) and 300 K (green) data. Similar behavior is reproduced at the II^*nd*^ set of thermal cycling also. In addition, spectral weight originating from the filled BCB states also is found to be enhancing in the vicinity of the E_*f*_ as shown in the inset. The trend under thermal cycling shown by the EDC spectra of N_2_ case (Fig. 6(k)) qualitatively matches with that of the Ar exposure case, whereas, the EDC plots of the O_2_ exposure case (Fig. 6(l)) show an opposite behavior. The small recovery of annealed 300 K (green) spectra with respect to the 77 K (red) in the II^*nd*^ set of cycle could possibly be a signature of incomplete desorption of the O_2_ gas.

It was reported that in the binary TI Bi_2_Se_3_ the DP moves by 116 meV from its initial position just after cleaving to a saturation value under the influence of BB^9^. Our own measurements also showed a shifting of ~0.1 eV in the time scale of 11:00 hr. after the sample cleaving in Bi_2_Se_3_. It is interesting to note that this movement is substantially lesser in comparison to that in BSTS where it is ~0.2 eV in a similar time scale and experimental conditions (Fig. 1 of Supplementary note). Recent studies on Bi_2_Se_3_ have shown that not only the extra charges at the surface but also its periodic re-arrangement inside the bulk creates a Coulomb potential of long range order contributing to the BB^31^. This unique property is inherent to the layered structure of Bi_2_Se_3_ where charge is accumulated and depleted at both ends of each QL. Introduction of additional elements (Sb and Te) in the QL of BSTS leads to an asymmetry in the structure of the QL compared to the Bi_2_Se_3_. The presence of Te atoms in addition to the Se atoms at the terminating faces of the QL in Bi_1.5_Sb_0.5_Te_1.7_Se_1.3_ have been observed in STM measurements^16^. This could possibly provide different screening to the surface charges compared to the Bi_2_Se_3_. So an oscillatory behavior of charge density could persist at larger distances inside the bulk region and result in a Coulomb potential of higher and longer range giving rise to stronger BB in BSTS in comparison to the Bi_2_Se_3_. Our experimental observations, stronger BB and lower DP position in BSTS compared to Bi_2_Se_3_, are consistent with the ARPES results on a similar composition Bi_1.5_Sb_0.5_Te_1.7_Se_1.3_ reported by Golden *et al*.^32^. These authors have also attributed the difference in effective screening of the adsorbate-induced surface charge to the variation in the temporal evolution of the SSBs between Bi_1.5_Sb_0.5_Te_1.7_Se_1.3_ and Bi_2_Se_3_ compounds. In addition, they suggested that different compositions of the terminated face between the two compounds could also influence the sticking process of the residual gas atoms thereby leading to different BB behaviour. This argument is supported by our first principles results where we find that the nature of the SSBs are different in Se and Te terminated slab geometries (Fig. 4). Besides this, another factor affecting the BB process could be the difference in the relaxation process of the exposed surface of the two compounds. However, in case of Bi_2_Se_3_ Hofmann *et al*. have ruled out any surface lattice relaxation from their LEED study^26^.

In conclusion, we discussed the results of our experimental studies using ARPES and first principles based Quantum Espresso band structure calculations and confirmed the non-trivial topology of the SSBs in BSTS. Our calculations show that the SSBs are sensitive to the atomic composition of the terminating surface and our experimental data shows that they have a partial 3D character. We have undertaken a detailed study of the shifting of the DP by the BB effect with elapse of time as well as adsorption of gases after the crystal cleaving. We find that under the BB effect the DP in BSTS shifts by more than two times compared to that in Bi_2_Se_3_ to reach a saturation. Our results suggest that the stronger BB in BSTS could be due to the difference in screening of the surface charges because of the different compositions of the QLs of the two compounds. From the MDCs of the ARPES data we obtained an energy dispersion relation showing the warping strength of the Fermi surface in BSTS to be intermediate between those found in Bi_2_Se_3_ and Bi_2_Te_3_ and also is tunable by the ratio of chalcogen/pnictogen atoms. Further experiments reveal that the nature of the BB effects are highly sensitive to the exposure of the fresh surface to various gas species; Ar and N_2_ show signatures of hole doping while O_2_ shows those of electron doping. Our findings could have importance in the tuning of the DP in topological insulators especially the members of the BSTS family for technological applications.

## Methods

The high quality single crystal samples of BiSbTe_1.25_Se_1.75_ (BSTS) used in this study were grown by modified Bridgman method. Stoichiometric amounts of Bi(99.999%), Sb(99.999%), Te(99.999%) and Se(99.999%) were heated in evacuated quartz ampoules to a temperature of 1073 K followed by slow cooling. Large sized single crystals (~5 cm) were obtained which cleaved easily along planes normal to the c-axis. The ARPES experiments were carried out using the facilities associated with the BaDELPH beamline of ELETTRA synchrotron center, Italy, equipped with a SPECS Phoibos 150 hemispherical analyser. The photoemission spectra were collected on freshly cleaved (*in-situ* at 77 K) surfaces of crystals under a vacuum of the order of 4.0 × 10^−11^ mbar. In addition, ARPES data were taken by using our laboratory facility decked with a high flux GAMMADATA VUV He lamp (VUV5000) attached to a VUV monochromator (VUV5040) and a SCIENTA R3000 analyser. Fermi energies of the samples were calibrated by using a freshly evaporated Ag film on the sample holder. The total energy resolution estimated from the width of the Fermi edge, was about 27 meV for HeI excitation energy. The angular resolution was better than 1° in the wide-angle mode (8°) of the analyzer. All the measurements were performed inside the analysis chamber under a base vacuum of 3.0 × 10^−10^ mbar.

First-principles calculations were performed by using a plain wave basis set inherent in Quantum Espresso (QE)^33^. Many electron exchange-correlation energy was approximated by the Perdew-Burke-Ernzerhof (PBE) functional^34–36^. Fully relativistic ultrasoft^37^ and non relativistic norm conserving pseudopotentials were employed for spin-orbit-coulpled (SOC) and non SOC calculations respectively. Fine mesh of k-points with Gaussian smearing of the order 0.0001 Ry was used for sampling the Brillouin zone integration, and kinetic energy and charge density cut-off were set to 100 Ry and 450 Ry respectively. Surface state calculations on (111) plane were performed by using supercell structures of hexagonal unit cell consisting of six quintuple layer (QL) with a vacuum separation of ~26 Å. Experimental lattice parameters^14^ and atomic coordinates were relaxed under damped (Beeman) dynamics with respect to both ionic coordinates and the lattice vectors for all the structures.

### Data availability

The datasets generated during and/or analysed during the current study are available from the corresponding author on reasonable request.

## Electronic supplementary material

Supplementary Information
