# Supplementary material for: Band Structure of Topological Insulator BiSbTe1.25Se1.75
Source: Sci Rep. 2017 Jul 4;7:4567. doi: 10.1038/s41598-017-04985-y (PMC5496864; doi:10.1038/s41598-017-04985-y)
Supplement: Supplementary file 1 — Supplementary Information [file 41598_2017_4985_MOESM1_ESM.pdf]

Supplementary Information

Band Structure of Topological Insulator $BiSbTe_{1.25}Se_{1.75}$

H. Lohani, P. Mishra, A. Banerjee, K. Majhi, R Ganesan, U. Manju, D. Topwal, P. S. Anil Kumar and B.R. Sekhar

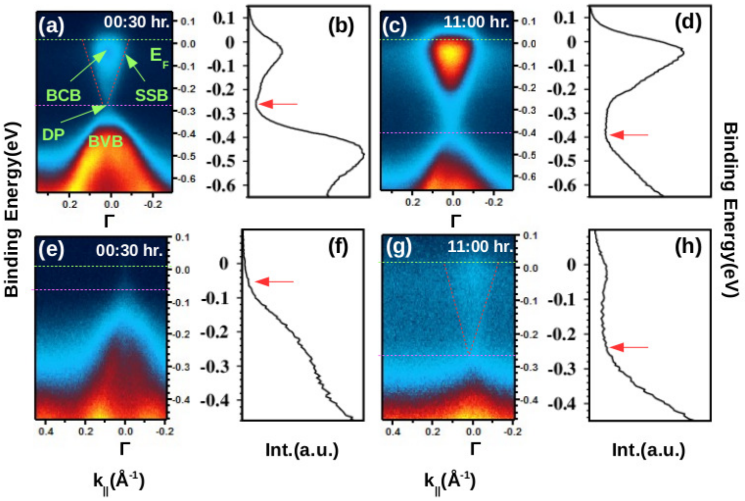

Figure 1: (a) and (c) show ARPES images of Bi_2Se_3 taken along the Γ-K' (15°) off set from perfect Γ-K) direction of the surface BZ and 0:30 hr. and 11:00 hr after the sample cleaving respectively. (b) and (d) represent energy density curves(EDCs) extracted from the corresponding images (a) and (c) respectively. (e) and (g) show ARPES images of BSTS taken along the Γ-M' (15°) off set from perfect Γ-M) direction of the surface BZ and 0:30 hr. and 11:00 hr. after the sample cleaving respectively. (f) and (h) correspond to the EDC plots extracted from the images (e) and (g) respectively. It may be noted that the BB induced changes are isotropic and hence do not depend on the off set in k-direction.

1 Comparison of band bending between BSTS and Bi_2Se_3.

Fig.1(a) and (c)show our ARPES images of Bi_2Se_3 taken along the Γ-K' (15° off set from perfect Γ-K) direction of the surface BZ using He-I (21.2 eV) photon energy while Fig.1(b) and (d) display energy density curves(EDC) extracted from the corresponding images (a) and (c) respectively. In the Fig.1(a) a cone shape intensity pattern is clearly visible and the two Dirac like dispersive SSBs are marked by red lines. These bands meet around BE $E_b \sim$ -0.28eV denoted as Dirac point (DP). Similarly, the region of bulk conduction band (BCB) and bulk valence band (BVB) are also marked. The DP moves towards higher BE as time elapses after the sample cleaving due to band bending (BB). This shift is $\sim$ 0.1 eV towards the higher BE in an 11:00 hour time scale as is clear from the Fig.1(c). These changes are also reflected in the EDCs as marked by red arrows in the Fig.1(b) and (d). Comparing this result with the ARPES images of BSTS shown in Fig.1(e) and (g) we find that the BB induces a shift of nearly two times indicating a stronger BB in BSTS compared to Bi_2Se_3. Since the SSBs and the position of DP is not clear in Fig.1(e) and (g) we have compared the position of the BVB maxima in their corresponding EDC plots Fig.1(f) and (h). We find the BVB(marked with red arrow) to be shifted by around 0.2 eV in case of BSTS in 11:00 hr. Experiments on both the samples were performed using a laboratory facility under same conditions.
